# Exposure to the Dutch Famine in Early Gestation and Cognitive Function and Decline in Older Age

**DOI:** 10.3390/nu15020293

**Published:** 2023-01-06

**Authors:** Aline Marileen Wiegersma, Amber Boots, Tessa J. Roseboom, Susanne R. de Rooij

**Affiliations:** 1 Epidemiology and Data Science, Amsterdam UMC, University of Amsterdam, Meibergdreef 9, Meibergdreef 9, 1105 AZ Amsterdam, The Netherlands; 2 Amsterdam Public Health Research Institute, Aging & Later Life, Health Behaviors & Chronic Diseases, 1007 MB Amsterdam, The Netherlands; 3 Amsterdam Reproduction and Development, 1105 AZ Amsterdam, The Netherlands; 4 Obstetrics and Gynaecology, Amsterdam UMC, University of Amsterdam, Meibergdreef 9, 1105 AZ Amsterdam, The Netherlands

**Keywords:** prenatal undernutrition, prenatal famine, cognitive function, cognitive decline, self-perceived cognitive problems

## Abstract

People exposed to the 1944–1945 Dutch famine in early gestation performed worse on a selective attention task at age 58 and reported more cognitive problems at age 72. We here hypothesized that undernutrition in early gestation is associated with poorer cognitive functioning in older age and a higher rate of cognitive decline. We tested this hypothesis in the Dutch famine birth cohort in men and women combined and separately. We assessed cognitive function using a Stroop-like, trail-making and 15-word task (at ages 68 and 74) and the Montreal cognitive assessment as well as self-perceived cognitive problems (at age 74) in 73 men (*n* = 34) and women (*n* = 39). We compared cognitive function and decline (change in cognitive function between age 68 and 74) between those exposed in early gestation and those not exposed (born before or conceived after the famine). Although in both men and women cognitive function declined from age 68 to 74, cognitive task scores and the rate of decline did not differ between those exposed or unexposed to famine. At age 74, men exposed to famine in early gestation more often reported cognitive problems, although this was not statistically different from unexposed men (OR 3.1 [95%CI 0.7 to 13.0]). We did not find evidence of increased cognitive decline after prenatal undernutrition. Selective participation and mortality may have hampered our ability to detect potential true effects. The self-perceived cognitive problems among men who had been exposed to famine in early gestation might be an indication of future dementia risk.

## 1. Introduction

The majority of brain development occurs during the prenatal period, with the largest part of neurogenesis occurring in the first half of gestation [1]. During this critical developmental phase, the brain is sensitive to the environment which may influence brain development with potentially lasting consequences for cognitive function across the lifespan [2].

Indeed, adverse prenatal factors, including air pollution and maternal stress, have been associated with poorer cognitive function in childhood as well as adulthood [3,4,5]. In addition to poorer cognitive function in early and adult life, there is evidence that adverse prenatal factors may increase the rate of cognitive decline that occurs with aging. Rodent studies have shown that adverse prenatal circumstances may accelerate cognitive decline and increase the vulnerability to developing Alzheimer’s disease-related pathologies [6]. In humans, markers for adverse prenatal circumstances, such as lower birth weight or being born in a high stroke incidence or high infant mortality area, have been associated with increased cognitive decline and dementia risk [7,8,9].

We have previously shown that men and women exposed to undernutrition in early gestation performed worse on a Stroop-like task administered during a stress protocol around age 58 in the Dutch famine birth cohort (DFBC), suggesting that prenatal famine exposure has lasting consequences for cognitive function. Performing the Stroop-like task requires selective attention and inhibition—cognitive abilities which are among the first to decline with age [10]. Therefore, our findings on the Stroop-like task may have been an early sign of accelerated cognitive aging [11]. Our subsequent findings, that men exposed to famine in early gestation reported more self-perceived cognitive problems at age 72 and more often consulted a healthcare professional for these problems, is in line with this hypothesis, as self-perceived cognitive problems have been associated with (future) cognitive decline and increased dementia risk [12,13,14,15]. Apart from worse performance on the Stroop-like task and poorer self-perceived cognitive function, we found that men exposed to famine in early gestation had worse brain perfusion, smaller total brain volumes, and increased BrainAGE (an MRI-based measure estimating clinical brain aging). These brain outcomes are potentially related to neurodegeneration and associated cognitive decline [16,17,18]. In addition, we observed patterns of altered resting-state functional connectivity at age 68, which in men were similar to patterns of network desegregation observed in brain aging [19]. Together, these neuroimaging studies suggest accelerated aging of the brain among men exposed to famine in early gestation [16,17,18,19].

Prenatal undernutrition may thus have led to accelerated cognitive and brain aging in men and women conceived during the Dutch famine. Similarly, studies that investigated prenatal exposure to the 1959–1961 Great Chinese Famine showed more cognitive impairment and a higher dementia prevalence among those exposed prenatally to famine [20,21,22,23]. However, the observed differences in cognition and brain size and function in the DFBC may have been present from early life as a result of hampered brain development and thereby not the result of accelerated aging [11,16,17,18,19].

In the current study, we aimed to test the hypothesis that exposure to the Dutch famine in early gestation is associated with worse cognitive function in older age and a higher rate of cognitive decline by studying cognitive function over time. Given our previous findings regarding sex-specific effects of prenatal famine exposure, we tested these hypotheses in the total group as well as in men and women separately.

## 2. Materials and Methods

### 2.1. Participants

Participants were a sample of men and women from the DFBC, which consists of 2414 individuals born alive as term singleton babies in the Wilhelmina Gasthuis (a teaching hospital in Amsterdam, the Netherlands) between 1 November 1943 and 28 February 1947 [24]. In 2012, a random subsample of the total eligible cohort (alive, living in the Netherlands and with a known address; *n* = 1307) was invited to participate in a study consisting of a home visit and MRI scan. In total, 151 persons agreed to be visited at home. A more detailed description of the selection procedure has been published before [17,25]. In 2019, we aimed to include all available participants from the 2012 study for a hospital visit encompassing extensive cognitive testing and a brain MRI scan. In the current study, we focused on the 73 participants who completed cognitive testing in both 2012 and 2019 (mean age 68 and 74 years). Two participants with dementia had been excluded as their scores on the cognitive tests were outliers and they did not complete all cognitive tests.

### 2.2. Exposure

Towards the end of World War II, a cascade of events led to a severe famine in the western part of the Netherlands [11,26]. Daily food rations dropped steeply, reaching a level below 1000 kilocalories per person on 26 November 1944 and improved relatively quickly after the Netherlands was liberated in May 1945. In the DFBC, three 16-week periods of exposure to undernutrition have been defined: individuals mainly exposed in late gestation (born between 7 January and 28 April 1945), mid-gestation (born between 29 April and 18 August 1945), or early gestation (born between 19 August and 8 December 1945) [24]. Individuals born in these periods were prenatally exposed to at least 13 weeks of famine, during which their mothers’ daily food ration contained on average fewer than 1000 kilocalories according to the official daily food rations for the general population of 21 years and older [27]. In previous DFBC studies, we observed the most negative health consequences after famine exposure in early gestation [24]. Therefore, only those exposed during early gestation were included in the current study [11]. Individuals born before (born before 7 January 1945) and conceived after the famine (born after 8 December 1945) were considered unexposed and acted as a control group.

### 2.3. Study Parameters

Maternal, pregnancy, and birth characteristics (e.g., maternal age and birth weight) were collected from medical birth records. We included the occupation (manual yes/no) of the head of the household as a measure of parental SES. Adult characteristics were collected from interview questions at ages 68 and 74. Level of education was defined as the highest level of finished schooling measured on a 7-point scale. Socioeconomic status (SES) was defined according to the International Index of Occupational Status 92, which is based on the participant’s or their partner’s (former) occupation, whichever status was highest [28].

### 2.4. Cognitive Function

Cognitive function was assessed with the Stroop-like task, trail-making task, and 15-word task at age 68 (at home) and age 74 (in the hospital). The Stroop-like task measures executive function, specifically selective attention and response inhibition, with a color-word incongruence task. The trail-making task also measures executive functioning, mainly, visual attention, task switching, and mental flexibility. The 15-word task measures verbal memory.

In addition to the three cognitive tasks that were repeated over time, we performed the standardized Montreal cognitive assessment (MoCA; multiple cognitive domains) and measured self-perceived cognitive problems at age 74 to obtain a more complete picture of the cognitive function of the participants at an older age [29,30]. Similar to these measurements performed at age 72, we assessed self-perceived cognitive problems with two questions, assessing if participants had problems with their memory, attention, and thinking and if they ever consulted a healthcare practitioner for these problems [12]. In addition, we used the Dutch version of the Cognitive Failures Questionnaire (CFQ) to measure self-perceived cognitive functioning in daily life [31]. For detailed information on cognitive testing and the rationale for including these tasks, see Supplementary Text S1.

### 2.5. Statistical Analyses

We performed linear and logistic regression analyses to compare cognitive outcomes (measured by cognitive tasks or self-reporting) between those exposed to famine in early gestation and the unexposed group at a mean age of 68 or 74. The Stroop-like task and trail-making task scores did not follow a normal distribution and commonly applied transformations (e.g., log transformations) did not normalize the scores. Therefore, we used ranked scores as the dependent variables in linear regression analyses to analyze these tasks. As the Stroop-like task, trail-making task, and 15-word task were measured at both ages 68 and 74, we additionally used generalized estimating equation models (GEE) using an exchangeable working correlation structure to examine changes over time, thereby accounting for the dependency of repeated measurements within persons. We included interaction terms for ‘exposure × time’ to evaluate if the rate of cognitive decline differed among the exposed and unexposed groups. In addition to the crude analyses, we adjusted the models for covariates (sex and parental SES). We additionally performed the analyses separately for men and women.

To evaluate participation bias, we compared baseline characteristics in the complete DFBC (*n* = 2414) between those included and not included in the current study. Furthermore, for those participating in 2002 (*n* = 717), we compared cognitive scores and SES collected around age 58 between those included and not included in the current study.

We used IBM SPSS statistics version 28.0 to perform the statistical analyses. We considered differences to be statistically significant if *p*-values were ≤0.05.

## 3. Results

Of the 73 participants (34 men and 39 women) included in the study, 25 were exposed to famine in early gestation. We did not detect statistically significant differences in maternal and birth characteristics according to prenatal famine exposure (exposed versus unexposed). When comparing those born before the famine with those conceived after the famine, we found that those born before the famine had a statistically significant smaller head circumference at birth (Table 1).

We did not detect differences in performance on any of the cognitive tasks at ages 68 or 74 between participants exposed to famine in early gestation and unexposed participants in the total group (Table 2) or men and women separately (Table 3). Adjusting for covariates did not alter the results.

On average, participants scored worse on the cognitive tasks around age 74 compared with age 68 years, which was statistically significant for the trail-making and 15-word summary tasks (*p* < 0.001; Table 4). However, in the GEE models combining men and women, the interaction terms for ‘exposure × time’ were not statistically significant (*p* > 0.05), indicating no altered rate of cognitive decline for the exposed compared with the unexposed groups. Sex-specific analyses showed that the rate of decline on the trail-making task was lower (i.e., a smaller increase in time needed to complete the tasks) for women exposed to famine in early gestation compared with unexposed women, this difference was statistically significant for task B (Task A: B −5.9 [95%CI −12.3 to 0.6]; Task B: B −40.1 [95%CI −65.5 to −14.8]; Table 5). In contrast, men exposed to famine in early gestation had a larger decline in the trail-making task from age 68 to 74, which was close to statistical significance in task A (Task A: B 10.5 [95%CI −0.5 to 21.6]; task B: B 10.9 [95%CI −15.2 to 37.0]).

The prevalence of self-perceived cognitive problems did not significantly differ between those exposed to famine in early gestation and those unexposed across all participants. Sex-specific analyses showed no difference for women (OR 0.9 [95%CI 0.2 to 3.7]. Exposed men reported more self-perceived cognitive problems compared with unexposed men, although this difference was not statistically significant (OR 3.1 [95% CI 0.7 to 13.0]; Table 3).

Regarding the evaluation of participation bias, birth weight did not differ among cohort members included or not included in the current study. Head circumference was smaller for those born before the famine included in the current study compared with those not included. For individuals who participated in 2002, men and women exposed to famine in early gestation included in the current study had higher scores on the Stroop-like task (*n* = 65; mean score 48.4 compared with 33.1, *p* = 0.12) and a higher SES (*n* = 65; mean 52.6 compared with 43.7, *p* = 0.01) at age 58 compared with those not included in the current study. Included men and women conceived after the famine also had higher scores on the Stroop-like task at age 58 compared with those not included in the current study (*n* = 65; mean 68.3 compared with 49.3, *p* = 0.01).

## 4. Discussion

In the current study, we did not find evidence to support our hypothesis that exposure to famine in early gestation leads to worse cognitive function in older age and faster cognitive decline. Although the cognitive performance of the participants as a group declined over time, demonstrating the deterioration of memory and executive functions, the rate of cognitive decline did not differ for those exposed to famine in early gestation compared with those not prenatally exposed to the famine. In line with our previous findings, self-perceived cognitive problems were highest in men exposed to famine in early gestation; however, the difference compared with unexposed men was not statistically significant here.

In the current study, we did not detect differences in the Stroop-like task comparing men and women exposed to famine in early gestation with those unexposed, while in a previous study at age 58, participants from the DFBC exposed to famine in early gestation performed worse on the Stroop-like task [11]. An explanation for this discrepancy may be that the Stroop-like task conducted around age 58 was part of a psychological stress protocol, while the Stroop-like task at ages 68 and 74 was not. Performing the Stroop-like task under pressure may have added another level of complexity, resulting in an even larger demand for attentional resources than simply provoked by the task itself [32,33]. This may have unmasked differences in performance on the task that would go undetected under normal circumstances. The fact that the cognitive tasks were not part of a stress protocol at ages 68 and 74 may have reduced the detectability of group differences in selective attention. In line with this explanation are findings from a study by de Groot et al., which showed no overall association between prenatal famine exposure and performance on a Stroop task not conducted during a stress protocol at age 59 [34]. Alternative or additional explanations for the absence of an association between prenatal famine exposure and performance on the Stroop-like task in the present study are selective participation and selective mortality, which are explained in detail below.

Similar to the Stroop-like task, we did not detect differences in the trail-making task, 15-word task, or MoCA between those exposed or unexposed to famine in early gestation. Furthermore, although cognitive performance declined over time, the rate of decline did not differ between exposed and unexposed participants. Together, these findings do not support our hypothesis of accelerated cognitive aging after famine exposure in early gestation. Accelerated cognitive aging in those exposed to famine in early gestation was expected based on our previous findings showing more self-perceived cognitive problems, which may be predictive of future cognitive decline [12,13,14]. Furthermore, findings suggesting more rapid aging of the brain, especially in men exposed to famine in early gestation, would also indicate accelerated cognitive decline [16,17,18,19]. The discrepancy between expectations based on previous observations and the current results could be explained by two scenarios.

First, as cognitive decline and the risk of dementia increase with age, we could have been too early to detect differences in cognitive function between those prenatally exposed or unexposed to the famine in the current study. Studies have shown that cognitive tasks often do not detect cognitive problems at the moment that early signs of neurodegeneration become apparent in the brain and participants self-report cognitive problems in daily life, suggesting that cognitive tasks may not adequately capture the first stages of cognitive decline [13,14,35].

Secondly, in addition to having been too early to detect differences in cognitive function, selective participation of more healthy and cognitively fit exposed men and women may have prevented us from detecting an association between prenatal famine exposure and worse cognitive functioning. We have previously shown that women exposed to famine in early gestation had higher overall adult mortality compared with unexposed women, which may have resulted in stronger women participating in the present study [36]. This may explain the lower decline in performance on the trail-making task among exposed women compared with unexposed women. Furthermore, we have previously shown that men and women exposed to famine in early gestation more often rated their health as poor, had a more atherogenic lipid profile, and had a higher risk of coronary heart disease and diabetes [37,38,39,40,41]. Therefore, a relatively larger proportion of participants exposed during early gestation may not have been fit enough to visit the clinic compared with unexposed participants. This may have resulted in a larger proportion of exposed participants with relatively better health remaining in the study; who may also have better cognitive function, given the relationship between these outcomes [42]. At age 58, exposed individuals participating in the current study had higher Stroop-like scores compared with exposed individuals who did not participate in the current study, indicating that those with lower performance indeed have more often decided not to participate in the current study. Taken together, these findings suggest that participation bias may have prevented us from detecting differences in cognitive function that would have been present if selective participation had not occurred.

As self-perceived cognitive problems usually occur before cognitive decline can be detected on cognitive tasks and are predictive for future cognitive decline and dementia [13,14,15], measuring self-perceived cognitive problems may be a more sensitive method to detect subtle differences in cognitive function that are typical for the early stages of cognitive decline. We indeed observed more self-perceived cognitive problems in men exposed to famine in early gestation compared with unexposed men in the current study. Although the difference was not statistically significant, the effect size was in line with what we have observed before in a larger sample in the DFBC [12]. Due to undernutrition, fewer children were conceived during the famine [24,43]; therefore, the number of eligible participants exposed to famine in early gestation was limited. Together with decreased participation, this led to a relatively small sample size in the current study. This small sample size may have prevented us from detecting a statistically significant effect, and our results might point to a process of cognitive decline in exposed individuals.

## 5. Conclusions

We did not identify differences in cognitive functioning or the rate of cognitive decline among those exposed or unexposed to famine in early gestation. Selective participation and mortality may have hampered our ability to detect potential true effects. Corresponding to previous findings, men exposed in early gestation more often reported cognitive problems, possibly indicative of future cognitive decline and dementia.

## Figures and Tables

**Table 1:** General, maternal, pregnancy, birth and adult characteristics by timing of prenatal exposure to the Dutch famine.

	*n*	Born before	Exposure to Famine in Early Gestation	Conceived after	Total
**General characteristics**					
N	73	23	25	25	73
% Women	73	60.9	44.0	56.0	53.4
**Maternal and pregnancy characteristics**					
Pregnancy duration (days)	62	284 (10)	287 (11)	282 (14)	285 (11)
Maternal age at birth (years)	73	28.3 (5.5)	26.5 (6.7)	27.6 (6.1)	27.5 (6.1)
% Primiparous	73	21.7	44.0	36.0	34.2
% Manual occupation head of household	56	93.3	60.9	61.1	69.6
**Birth characteristics**					
Birth weight (g)	73	3337 (533)	3492 (507)	3357 (534)	3396 (522)
Head circumference (cm)	72	32.0 (1.3) *	32.9 (1.2)	33.0 (1.7)	32.6 (1.5)
**Adult characteristics**					
Level of education ^a^	73	4.8 (2.6)	4.9 (1.9)	4.4 (2.1)	4.7 (2.2)
SES	73	49.8 (12.8)	50.2 (13.8)	47.7 (16.1)	49.2 (14.2)
Age 2012 (years)	73	68.6 (0.4) **	67.3 (0.2	66.7 (0.4)	67.5 (0.8)
Age 2019 (years)	73	75.2 (0.5) **	74.0 (0.2)	73.4 (0.5)	74.2 (0.8)

Numbers represent frequencies (%) or means (SD). Asterisks represent *p*-values of linear and logistic regression analyses for those exposed to famine in early gestation compared with participants unexposed to famine in gestation, or, for individuals born before the famine, compared with individuals conceived after the famine. ^a^ Based on a 7-point scale; (1) Less than six years of primary school, (2) six years of primary school, (3) more than primary school, without an additional diploma, (4) craft school, (5) (pre-)secondary vocational education, (6) pre-university education, (7) higher professional education/university. * < 0.05, ** < 0.001.

**Table 2:** Associations between prenatal exposure to famine and cognitive outcomes compared with individuals born before or conceived after the famine around age 68 (2012) and 74 (2019).

Outcome	*n*	Born before (Reference)	Exposure to Famine in Early Gestation	Conceived after (Reference)	B (95%CI)	*p*-Value
	73	23	25	25		
**2012**						
**Stroop-like task ^a^**						
% Correct ^b^	73	54.0 (28.1)	61.1 (29.9)	53.3 (34.5)	^c^	0.41
Response time (s)	68	3.4 (0.2)	3.3 (0.6)	3.4 (0.7)	−0.1 (−0.4–0.2)	0.47
**Trail-making task ^a^**						
Trail A (sec) ^d^	73	34.0 (9.0)	32.0 (7.0)	31.0 (22.0)	^c^	0.67
Trail B (sec) ^d^	73	80.0 (30.0)	70.0 (28.0)	75.0 (35.0)	^c^	0.29
**15-word task**						
Summary 5 trials	73	32.8 (8.0)	32.7 (8.6)	30.4 (8.4)	1.2 (−2.9–5.3)	0.57
Recall	73	5.5 (3.4)	6.5 (2.6)	6.2 (2.2)	0.6 (−0.7–2.0)	0.38
**2019**						
**Stroop-like task ^a^**						
% Correct ^b^	73	50.4 (29.9)	53.0 (30.0)	48.5 (34.5)	^c^	0.51
Response time (s)	71	3.9 (0.5)	3.8 (0.5)	3.7 (0.6)	−0.0 (−0.3–0.2)	0.76
**Trail-making task ^a^**						
Trail A (sec) ^d^	73	45.0 (18.0)	38.0 (16.0)	40.0 (14.5)	^c^	0.62
Trail B (sec) ^d^	73	104.0 (58.0)	84.0 (37.0)	87.0 (106.0)	^c^	0.26
**15-word task**						
Summary 5 trials	73	29.7 (9.5)	29.2 (6.3)	26.3 (7.5)	1.3 (−2.6–5.1)	0.52
Recall	73	6.0 (2.6)	5.4 (2.0)	5.1 (2.4)	−0.1 (−1.3–1.0)	0.81
**MoCA**						
Summary score	73	22.9 (2.2)	23.5 (2.7)	23.4 (3.3)	0.3 (−1.1–1.7)	0.67
**Self-perceived cognitive problems**						
Problems with memory, attention and thinking	73	8 (34.8)	12 (48.0)	9 (36.0)	1.7 ^e^ (0.6–4.5)	0.30
Sought medical help	73	0	0	2 (8.0)	^f^	
CFQ score	73	26.5 (9.8)	28.7 (8.7)	26.9 (10.2)	2.0 (−2.7–6.7)	0.40

CFQ = Cognitive Failures Questionnaire (lower score means more failures). MoCA = Montreal Cognitive Assessment. ^a^ Analysis was based on rank scores. ^b^ As the Stroop-like scores followed a bimodal distribution, including a flat center including empty areas, median scores did not accurately reflect the central tendency. We therefore used mean (SD) to describe the Stroop-like results even though the data was not normally distributed. ^c^ Estimates were not meaningful as they are based on ranked data. ^d^ Data given as median (IQR) instead of mean (SD). ^e^ Odds ratio based on logistic regression. ^f^ Too few cases to perform statistical analyses.

**Table 3:** Associations between prenatal exposure to famine and cognitive outcomes compared with individuals born before or conceived after the famine around age 68 (2012) and 74 (2019) by sex.

Outcome	*n*	Born before (Reference)	Exposure to Famine in Early Gestation	Conceived after (Reference)	B (95%CI)	*p*-Value
**2012**						
**Men**		9	14	11		
**Stroop-like task ^a^**						
% Correct ^b^	34	50.2 (10.7)	62.6 (8.0)	66.2 (10.0)	^c^	0.99
Response time (s)	32	3.2 (0.1)	3.2 (0.1)	3.3 (0.2)	−0.1 (−0.5–0.3)	0.65
**Trail-making task ^a^**						
Trail A (sec) ^d^	34	33.0 (9.0)	31.0 (8.0)	31.0 (22.0)	^c^	0.54
Trail B (sec) ^d^	34	81.0 (35.0)	70.5 (32.0)	59.0 (37.0)	^c^	0.97
**15 word task**						
Summary 5 trials	34	28.3 (2.1)	29.5 (2.1)	27.0 (2.6)	1.9 (−3.5–7.3)	0.48
Recall	34	4.2 (0.9)	5.6 (0.6)	5.1 (0.7)	0.9 (−0.8–2.5)	0.29
**Women**		14	11	14		
**Stroop-like task ^a^**						
% Correct ^b^	39	56.5 (7.0)	59.1 (9.4)	43.2 (8.9)	^c^	0.42
Response time (s)	36	3.5 (0.0)	3.5 (0.2)	3.5 (0.2)	−0.0 (−0.4–0.4)	0.87
**Trail-making task ^a^**						
Trail A (sec) ^d^	39	35.0 (13.0)	35.0 (7.0)	31.0 (22.0)	^c^	0.98
Trail B (sec) ^d^	39	78.0 (37.0)	70.0 (25.0)	77.0 (27.0)	^c^	0.24
**15 word task**						
Summary 5 trials	39	35.7 (2.1)	36.8 (2.4)	33.0 (2.1)	2.5 (−3.2–8.1)	0.38
Recall	39	6.4 (1.0)	7.6 (0.9)	7.1 (0.4)	0.9 (−1.1–2.9)	0.36
**2019**						
**Men**		9	14	11		
**Stroop-like task ^a^**						
% Correct ^b^		57.0 (9.6)	54.7 (6.7)	55.9 (11.2)	^c^	0.86
Response time (s)		3.8 (0.2)	3.8 (0.1)	3.7 (0.2)	0.0 (−0.3–0.4)	0.80
**Trail-making task ^a^**						
Trail A (sec) ^d^		34.0 (15.5)	38.5 (21.5)	30.0 (14.0)	^c^	0.24
Trail B (sec) ^d^		85.0 (38.0)	85.0 (68.0)	87.0 (117.0)	^c^	0.76
**15 word task**						
Summary 5 trials		27.0 (2.3)	28.2 (1.4)	25.4 (2.5)	2.1 (−2.6–6.8)	0.37
Recall		4.7 (0.8)	5.0 (0.5)	4.5 (0.8)	0.5 (−1.2–2.1)	0.58
**MoCA**						
Summary score	34	23.9 (0.5)	23.1 (0.7)	24.5 (0.8)	−1.1 (−2.8–0.5)	0.18
**Self-perceived cognitive problems**						
Problems with memory, attention and thinking	34	4 (44.4)	8 (57.1)	2 (18.2)	3.1 ^e^ (0.7–13.0)	0.12
Sought medical help	34	0	0	1 (9.1)	^f^	
CFQ score	34	28.3 (3.3)	29.8 (2.2)	23.6 (3.8)	4.0 (−3.3–11.4)	0.27
**Women**						
**Stroop-like task ^a^**						
% Correct ^b^		46.2 (8.2)	50.8 (11.0)	42.6 (8.7)	^c^	0.61
Response time (s)		3.9 (0.1)	3.7 (0.2)	3.8 (0.1)	−0.1 (−0.5–0.3)	0.54
**Trail-making task ^a^**						
Trail A (sec) ^d^		46.5 (27.3)	36.0 (16.0)	41.0 (11.5)	^c^	0.09
Trail B (sec) ^d^		123.0 (112.0)	83.0 (13.0)	97.5 (98.0)	^c^	0.05
**15 word task**						
Summary 5 trials		31.4 (2.9)	30.5 (2.3)	27.1 (1.9)	1.2 (−5.1–7.5)	0.70
Recall		6.8 (0.7)	5.8 (0.6)	5.6 (0.6)	−0.4 (−2.0–1.3)	0.67
**MoCA**						
Summary score	39	22.3 (0.6)	24.0 (0.9)	22.6 (0.9)	1.5 (−0.6–3.7)	0.16
**Self-perceived cognitive problems**						
Problems with memory, attention and thinking	39	4 (28.6)	4 (36.4)	7 (50.0)	0.9 ^e^ (0.2–3.7)	0.89
Sought medical help	39	0	0	1 (7.1)	^f^	
CFQ score	39	25.3 (2.6)	27.3 (2.8)	29.4 (1.9)	−0.1 (−6.5–6.4)	0.98

CFQ = Cognitive Failures Questionnaire (lower score means more failures). MoCA = Montreal Cognitive Assessment. ^a^ Analysis was based on rank scores. ^b^ As the Stroop-like scores followed a bimodal distribution, including a flat center including empty areas, median scores did not accurately reflect the central tendency. We therefore used mean (SD) to describe the Stroop-like results even though the data was not normally distributed. ^c^ Estimates were not meaningful as they are based on ranked data. ^d^ Data given as median (IQR) instead of mean (SD). ^e^ Odds ratio based on logistic regression. ^f^ Too few cases to perform statistical analyses.

**Table 4:** Generalized estimation equation (GEE) models with ‘exposure × time’ interaction terms comparing cognitive outcomes between participants prenatally exposed to famine and unexposed participants.

	Mean/Median Difference ^a^ 2012	Mean/Median Difference ^a^ 2019	GEE Model	
			Estimate (B) (95%CI)	*p*-Value
**15-word task**				
Summary				
Prenatal famine	1.18	1.26	1.2 (-2.9-5.2)	0.57
Time			**−3.6 (−5.4–1.8)**	**<0.001**
Interaction ^b^			0.1 (−3.4–3.5)	0.96
Recall				
Prenatal famine	0.61	−0.14	0.6 (−0.7–1.9)	0.36
Time			−0.4 (−1.1–0.3)	0.30
Interaction ^b^			−0.7 (−1.9–0.4)	0.19
**Trail-making task (higher score is worse)**				
Test A				
Prenatal famine	−1	−2	−2.9 (−7.3–1.4)	0.19
Time			**6.4 (3.1–9.7)**	**<0.001**
Interaction ^b^			2.1 (−5.0–9.1)	0.57
Test B				
Prenatal famine	−7.5	−18	−10.5 (−25.5–4.5)	0.17
Time			**36.8 (20.5–53.0)**	**<0.001**
Interaction ^b^			−15.6 (−35.4–4.23)	0.12
**Stroop-like task**				
Prenatal famine	7.41	3.57	7.4 (−7.0–21.9)	0.32
Time			−4.3 (−11.8–3.3)	0.27
Interaction ^b^			−3.8 (−19.5–11.8)	0.63

Statistically significant results are depicted in bold. ^a^ Mean or median (for the trail-making task) difference between the scores for those exposed or unexposed prenatally to the famine at age 68 (T1) and age 74 (T2). ^b^ Interaction term for ‘exposure × time’ to evaluate if the rate of cognitive decline differed among the exposed and unexposed groups.

**Table 5:** Generalized estimation equation (GEE) models with ‘exposure × time’ interaction terms comparing cognitive outcomes between participants prenatally exposed to famine and unexposed participants by sex.

	Mean/Median Difference T1 ^a^	Mean/Median Difference T2 ^a^	GEE Model	
			Estimate (B) (95%CI)	*p*-Value
**Men**				
**15-word task**				
Summary				
Prenatal famine	1.90	2.11	1.9 (−3.2–7.0)	0.47
Time			−1.5 (−3.7–0.7)	0.18
Interaction ^b^			2.0 (−3.8–4.2)	0.92
Recall				
Prenatal famine	0.87	0.45	0.9 (−0.6–2.4)	0.26
Time			−0.2 (−1.1–0.8)	0.77
Interaction ^b^			−0.4 (−2.0–1.2)	0.60
**Trail-making task (higher score is worse)**				
Task A				
Prenatal famine	−1.00	5.50	−2.7 (−9.2–3.9)	0.42
Time			1.2 (−3.1–5.6)	0.58
Interaction			10.5 (−0.5–21.6)	0.06
Task B				
Prenatal famine	−8.50	−10	−6.7 (−33.0–19.7)	0.62
Time			**20.3 (0.1–40.5)**	**0.05**
Interaction			10.9 (−15.2–37.0)	0.41
**Stroop-like task**				
Prenatal famine	3.64	−1.68	3.6 (−17.0–24.3)	0.73
Time			−2.6 (−14.9–9.7)	0.68
Interaction ^b^			−5.3 (−28.0–17.4)	0.65
**Women**				
**15-word task**				
Summary				
Prenatal famine	2.46	1.21	2.5 (−2.9–7.8)	0.37
Time			**−5.1 (−7.5–2.7)**	**<0.001**
Interaction ^b^			−1.3 (−6.6–4.1)	0.64
Recall				
Prenatal famine	0.92	−0.36	0.9 (−1.0–2.8)	0.34
Time			−0.5 (−1.5–0.4)	0.28
Interaction ^b^			−1.3 (−2.7–0.2)	0.08
**Trail-making task (higher score is worse)**				
Task A				
Prenatal famine	0.50	−7.50	−2.8 (−8.5–2.9)	0.34
Time			**10.0 (5.8–14.3)**	**<0.001**
Interaction ^b^			−5.9 (−12.3–0.6)	0.08
Task B				
Prenatal famine	−7.00	−34.50	−14.7 (−30.7–1.4)	
Time			**48.5 (25.6–71.4)**	**<0.001**
Interaction ^b^			**−40.1 (−65.5–14.8)**	**0.002**
**Stroop-like task**				
Prenatal famine	9.23	6.35	9.2 (−11.4–29.9)	0.38
Time			−5.4 (−15.0–4.2)	0.27
Interaction ^b^			−2.9 (−24.5–18.8)	0.79

Statistically significant results are depicted in bold. ^a^ Mean or median (for the trail-making task) difference between the scores of those exposed or unexposed prenatally to the famine at age 68 (T1) and age 74 (T2). ^b^ Interaction term for ‘exposure × time’ to evaluate if the rate of cognitive decline differed among the exposed and unexposed groups.

## Data Availability

Data described in the manuscript, code book, and analytic code will be made available upon reasonable request.

## Supplementary Materials

The following supporting information can be downloaded at: https://www.mdpi.com/article/10.3390/nu15020293/s1, Text S1: Detailed information on the cognitive tests used in the current study.
