# Supplementary material for: Exposure to the Dutch Famine in Early Gestation and Cognitive Function and Decline in Older Age
Source: Nutrients. 2023 Jan 6;15(2):293. doi: 10.3390/nu15020293 (PMC9867093; doi:10.3390/nu15020293)
Supplement: Supplementary file 1 [file nutrients-15-00293-s001.zip › nutrients-1997630-supplementary.pdf]

nutrients

Supplementary text S1: Detailed information on the cognitive tests used in the current study

Exposure to the Dutch famine in early gestation and cognitive function and decline in older age

Wiegersma et al.

Cognitive function

Stroop-like task. The Stroop-like task measures executive function, specifically selective attention and response inhibition, with a color-word incongruence task. The task was computerized and took about five minutes. Color words were randomly presented in one of four different ink colors as a target (i.e., the word "red" printed in blue). The color word matching the target ink color had to be selected from four answer options at the bottom of the screen that were presented in different (incongruent) ink colors. Targets and answer options could be both congruent (i.e. color word matching the ink color) or incongruent (i.e. color word in a different ink color). Participants had five seconds to select the correct answer and received feedback when selecting the wrong answer. Both the average response time before selecting an answer and the amount of correct and incorrect answers was automatically recorded.

Trail-making task. The trail-making task also measures executive functioning. Specifically, this task requires visual attention, task switching and mental flexibility. The task consisted of two rounds (A and B) both preceded by a trial round. In both rounds, 25 dots had to be connected with a pen without lifting it. In round A, numbers had to be connected in ascending order. In round B, numbers and letters had to be connected in ascending order, alternating between numbers and letters. Participants were instructed to perform the task as fast as possible while maintaining accuracy. Mistakes were pointed out by the researcher and had to be corrected by the participant. The time needed to finish round A and B was separately recorded in seconds with a maximum of five minutes for either round.

15-word task. The 15-word task measures verbal memory. Participants had to listen to the same recording of 15 words in 5 consecutive rounds. After each round, participants had to recite as many words as they could remember. After 20 minutes, participants were asked to recall as many words as possible. Participants were not informed about the upcoming recall task.

Montreal Cognitive Assessment (MoCA). The MoCA measures multiple cognitive domains including executive functioning and memory [29]. We used the authorized Dutch translation of the MoCA (version 8.1). The MoCA consists of eight domains: Visuospatial/executive, naming, memory, attention, language, abstraction, delayed recall and orientation. All MoCA items add up to a maximum of 30 points. We used unadjusted MoCA scores (i.e., not adding a point for individuals with twelve years of education or less). The MoCA Clinic and Insitute granted permission to use the MoCA test and two researchers completed a mandatory online training to receive certification to conduct and score the MoCA test (AMW and AB). All tests were administrated and scored by one researcher (AMW), and the scores were checked by a second researcher (AB).

Self-perceived cognitive problems. Self-perceived cognitive problems were assessed with two questions which were also included in the 2018 survey of the DFBC at age 72 [12]. One question was selected from The Older Persons and Informal Caregivers Survey – Minimum DataSet (TOPICS-MDS) validated questionnaire (version before 2017) [12]. Participants had to select the answer that best described their health at that time: "1) I have no problems with my memory, attention and thinking; 2) I have some problems with my memory, attention and thinking; 3) I have severe problems with my memory, attention and thinking." A second question was adapted from the TOPICS-MDS (version 2017): "Did you consult a doctor or other healthcare practitioner for problems with your memory, attention and thinking in the past 12 months?" [12]. In addition to these questions, we used the Dutch version of the Cognitive Failures Questionnaire (CFQ) to measure subjective cognitive functioning in daily life [31]. Participants had to rate how often they made 25 common mistakes on a 5-point Likert scale.

Rationale for including these cognitive tasks. The tasks included in the current study are commonly used for measuring cognitive function and easy to set-up. Furthermore, as described above, the included tasks measure different domains of cognitive function, thereby providing a picture of overall cognitive functioning. The Stroop-like task was

specifically included as it measures cognitive functions that are among the first to decline with aging [10]. Furthermore, in a previous study in the Dutch famine birth cohort, men and women exposed to famine in early gestation performed worse on the Stroop-like task compared to the unexposed group [11]. In addition to tasks measuring specific cognitive domains we aimed to include a task measuring overall cognitive function. The Mini-Mental State Examination (MMSE) and MoCA are both commonly used in the clinic to detect mild cognitive impairment and dementia. In the current study we included the MoCA as this task is reported to be more sensitive for the detection of early cognitive problems [30].
